# Waveguide model of the hearing aid earmold system

**DOI:** 10.1186/1746-1596-1-6

**Published:** 2006-05-11

**Authors:** Grzegorz Szwoch, Bozena Kostek

**Affiliations:** 1 Gdansk University of Technology, Multimedia Systems Department, Narutowicza 11/12, 80-952 Gdansk, Poland; 2 Center of Excellence PROKSIM, Institute of Physiology and Pathology of Hearing, Pstrowskiego 1, 01-943 Warsaw, Poland

## Abstract

**Background:**

The earmold system of the Behind-The-Ear hearing aid is an acoustic system that modifies the spectrum of the propagated sound waves. Improper selection of the earmold system may result in deterioration of sound quality and speech intelligibility. Computer modeling methods may be useful in the process of hearing aid fitting, allowing physician to examine various earmold system configurations and choose the optimum one for the hearing aid user.

**Methods:**

In this paper, a computer model adequate for this task is proposed. This model is based on the waveguide modeling method. The waveguide model simulates the propagation of sound waves in the system of cylindrical tubes. Frequency response of the hearing aid receiver is simulated in the model and the influence of the ear canal and the eardrum on the earmold system is taken into account. The model parameters are easily calculated from parameters of a physical hearing aid system. Transfer function of the model is calculated and frequency response plots are obtained using the Matlab system.

**Results:**

The frequency response plots of the earmold model were compared to the measurement plots of the corresponding physical earmold systems. The same changes in frequency responses caused by modification of length or diameter of a selected waveguide section, are observed in both measurement data of a real earmold system and in computed model responses.

**Conclusion:**

Comparison of model responses obtained for various sets of parameters with measurement data proved that the proposed model accurately simulates the real earmold system and the developed model may be used to construct a computer system assisting the physician who performs earmold system fitting.

## Background

The number of hearing impaired individuals who need to use hearing aids is rapidly increasing nowadays. Proper tools for optimum hearing aid fitting are needed, allowing physician to choose and tune hearing aid most suitable to the user's needs in a short time. Methods for selecting amplification and compression characteristics in order to compensate hearing loss and improve speech intelligibility are now well developed. However, one aspect of the fitting process that is often underestimated is the design of the earmold system of a hearing aid. In the case of the miniaturized hearing aids (In-The-Ear and In-The-Canal) this problem is of less importance, because the earmold system is very simplified and it does not significantly affect sound quality. However, many individuals still need to use a BTE (Behind-The-Ear) hearing aid type, especially when high amplification is needed, that would cause acoustic feedback in miniaturized hearing aids, or if the hearing aid user is less technically skilled or manually impaired. The earmold system of BTE hearing aids, conducting sound waves from the receiver of the hearing aid to the ear canal of the hearing impaired person, is fairly complex [1,2]. Improper selection of the earmold system may deteriorate sound quality and, as a consequence, decrease speech intelligibility.

Physicians who perform the fitting process of a hearing aid, often concentrate only on amplification and compression parameters, neglecting the need of proper earmold system design. The choice of earmold system elements is reduced to testing of several earmolds and tubings and fitting ones that do not produce acoustic feedback and give satisfactory sound quality, as assessed subjectively by a hearing aid user. However, there is a wide range of possible earmold system selections, differing in physical sizes and material. Various earmold systems have different frequency characteristics [3]. If the proper tool was available allowing physicians to design the earmold system having frequency characteristics that are adequate for a person with a given hearing loss, they would be able to select the optimum earmold system. Thus, both electronic and earmold part of the hearing aid would be better fitted to the needs of a hearing impaired person [4,5].

Although computer modeling methods are widely used in many applications, only a few reports on modeling of the hearing aid earmold system are found in literature [6,7] and these models are not currently used in practice. The proposed models of the earmold system were designed as a combination of lumped elements and transmission lines. The main problem with this approach is the proper choice of model parameters, which in many cases has to be performed using measurements of the real earmold system. Moreover, the mathematical description of the model is fairly complex, so practical implementation of such a model is problematic.

In this paper, the waveguide model of this system is proposed. This model may be a part of the computer system allowing physician to design the earmold system having desired acoustic properties, based on the hearing loss characteristics of the hearing aid user. In this way, it would be possible to perform the first stage of earmold system fitting without the presence of a hearing impaired person. The second stage of fitting would start with testing of pre-designed earmold system. The waveguide model of the hearing aid earmold system and results of experiments are discussed in details in the following parts of this paper.

## Methods

### Waveguide model of the earmold system

The computer simulation technique has been developed by Smith [8] in late 80s at Stanford University. This method is called *waveguide modeling *and it has been successfully used in modeling musical instruments, allowing one to perform *waveguide sound synthesis *using the models [9,10]. The aim of digital waveguide modeling is to design a discrete-time model that behaves similarly to a physical system. So far, the waveguide modeling method has not been used to model earmold system of a BTE hearing aid. Acoustically, the earmold system of the BTE hearing aid is a duct consisting of tubes, allowing the propagation of sound waves from the hearing aid receiver to the ear canal [2]. The earmold system is typically divided into three parts. An *earhook*, made of hard plastic, protects microphone and receiver of the hearing aid from physical damages. A *tubing *is a long and narrow elastic tube, which connects earhook to the earmold. An *earmold *is inserted into the ear canal and its shape is anatomically fitted to the pinna and ear canal of the hearing aid user. The duct, consisting of earhook canal, tubing and earmold canal, may be represented as a set of cylindrical tubes. If sections of the duct have conical shape, a set of cylindrical sections only approximates the shape of the duct.

**Figure 1:**
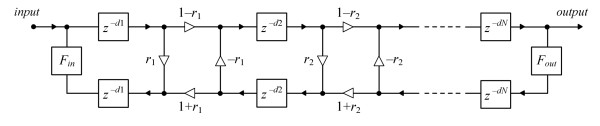
The waveguide model of the earmold system of the BTE hearing aid; *r *denotes reflection coefficient, *d *– delay line length.

The modeling of wave propagation in cylindrical tubes may be easily performed using the waveguide method, as described further on in this paper. This method models only one-dimensional wave propagation, no transversal modes may occur in the modeled system. In the cylindrical tube this condition is valid as long as frequency does not exceed critical value given by [10]:



where *c *is the velocity of wave propagation and *a *is the radius of cylindrical tube. In the hearing aid, the frequency range is usually limited due to the properties of the receiver.^1 ^Assuming that *f*_*c *_is equal to 11.025 kHz (which is half the sampling rate used in experiments described later in this paper) and *c *= 343 m/sec, the maximum allowed radius *a *calculated using Eq. 1.1 is 9.111 mm. This value is not exceeded in practically used earmold system elements, hence the waveguide modeling method may be applied to the earmold system of a hearing aid. The system of tubes fulfilling Eq. 1.1 will be called the *waveguide*. The waveguide model of a set of cylindrical tubes is shown in Fig. 1[5][11]. Each cylindrical tube is modeled as a pair of delay lines and the length of each delay line is a function of the length of the cylindrical tube.

In order to accurately model the influence of receiver on the earmold system, the model of the receiver itself has to be developed and the acoustic impedance of the receiver should be used to calculate the transfer function of the input reflection filter *F*_*in *_in the waveguide model of the earmold system. However, the problem of designing the accurate model of a hearing aid receiver is fairly complex. The specialized hearing aid receivers differ in structure from typical audio earphones, so the models developed for "large" receivers cannot be applied directly. Moreover, specifications published by receiver manufacturers do not provide all data necessary to design such a model. In most publications concerning the hearing aid modeling, these data were obtained by measurements [6].

In the computer model described in this paper, the main topic is the modeling of the hearing aid earmold system. The model of the receiver is mainly needed to simulate the low-pass character of this element and to take into account any resonances (local maxima) occurring in its frequency response. Therefore, in the described model the receiver is simulated in the simplified way. The frequency response of a given receiver type is determined, either by measuring the receiver or by approximating the plot of its frequency response. The response of the receiver is then multiplied by the frequency response of the earmold system model.

Any such frequency response of the receiver may be used. In the experiments described later in this paper, the measurement data of the BK1600 Knowles receiver, published in literature [12], were used. These data were interpolated in order to obtain continuous frequency response function of the receiver. The impedance of the receiver was not used in calculation of the model parameters and the input reflection filter *F*_*in *_was replaced by a constant coefficient *r*_0_.

### Ear canal and eardrum simulation

The output of the earmold system of a hearing aid is connected to the outer ear canal and the eardrum. In order to simulate the interactions between the earmold system and the ear canal with eardrum, the impedance of the latter may be used to calculate the transfer function of the output reflection filter *F*_*out *_in the model. In the model described in this paper, another method was used, in which the ear canal and the eardrum were modeled separately.

The length and shape of the ear canal are unique for each individual. However, a sufficiently accurate model of the ear canal valid for frequencies below 8 kHz is a cylindrical tube of 7.5 mm in length and 22.5 mm in diameter [13]. The useful frequency range in hearing aids rarely exceeds 8 kHz, mainly due to the deficiencies of the receiver, so that this model is adequate for this application. The ear canal is thus modeled by adding another section to the model of earmold system, described earlier. The length and diameter values specified above were used in the experiments, it is, however, possible to introduce other values. It should be, however, remembered that the length of the ear canal should be decreased by subtracting the length of earmold canal, which is inserted into the ear canal.

In order to model the acoustic properties of the eardrum, its acoustic impedance has to be known. Ideally, the impedance should be measured in a patient. In the experiments described in this paper, the averaged frequency characteristics of eardrum impedance, obtained in more than 20 studies was used [14]. It is important to note that these studies were performed on healthy subjects and the eardrum impedance of a hearing impaired person may be different. The tabulated study results were interpolated in order to obtain continuous complex function of eardrum impedance vs. frequency. This function was used to calculate the transfer function of the output reflection filter *F*_*out *_in the model.

### Vents simulation

When earmold is placed in the ear canal, it closes (occludes) the canal, causing raise in pressure inside the ear canal and unnecessarily amplifying the low frequency components of sound signal. This phenomenon is called *occlusion effect*. In order to compensate this effect, venting canals, often called *vents*, are drilled in the earmold, allowing pressure inside and outside the ear canal to equalize [1,2].

In the waveguide model, vents may be modeled in the same way that finger holes in musical instruments are modeled in waveguide synthesis [14]. A special form of the scattering junction is needed, which models the connection of three cylindrical tubes [10]. When the earmold system with typical parallel vent is modeled, one of these tubes is the earmold canal, second – ear canal, third – the vent. The reflection of the wave at the termination of the vent is modeled using the *vent reflection filter F*_*v*_(*z*). The transfer function of the vent reflection filter is given by formula [15]:



where coefficient *a *is given by:



*f*_*S *_is sampling frequency, *c *is wave velocity, *l*_*v *_is effective vent length equal to [16]:



*L*_*v *_is the physical length of the vent, *r*_*v *_is vent diameter and *r*_*t *_is tube diameter. The vent reflection filter *F*_*v*_(z) is an all-pass filter.

## Results and discussion

The waveguide model of the hearing aid earmold system, including the receiver, ear canal with eardrum and, optionally, vents, was implemented on a personal computer using the MATLAB system. A set of procedures were written by the authors in the internal MATLAB programming language. The system allows one to calculate and to plot frequency responses of the model. The system also enables altering length and diameter of each model section, and, in addition, comparing plots of transfer function for two sets of model parameters. It is also used to process sound files using the designed frequency response and examine results of this processing. The frequency responses of the model developed were calculated and plotted for varying length and diameter of each section of the waveguide. In order to test the validity of the model, changes in frequency response plot of the model caused by the change of length or diameter of the chosen waveguide section were compared to the measurement data, published by the earmold system manufacturers [3]. The assumption was made that if the designed model properly simulates physical earmold system, changes in the frequency response plot should correspond to the changes in relevant measurement data.

The waveguide model used in experiments consisted of four cylindrical sections. In the reference model, which simulates the typical earmold system, the following values were used: earhook length 17 mm, earhook diameter 1.8 mm, tubing length 45.8 mm, tubing diameter 2 mm, earmold canal length 10 mm, earmold canal diameter 2.4 mm, ear canal length 22.5 mm, ear canal diameter 7.5 mm. The vent was not used. Instead of the input reflection filter *F*_*in*_, a constant coefficient *r*_0 _was used. Its value was experimentally chosen as *r*_0 _= -0.5 in order to simulate energy loss in the model. The transfer function of the output reflection filter *F*_*out *_was calculated using the average eardrum impedance characteristics, as previously discussed. The frequency response of the model was multiplied by the interpolated frequency response of a typical receiver (Knowles BK1600). The sampling frequency 22050 kHz was chosen.

**Figure 2:**
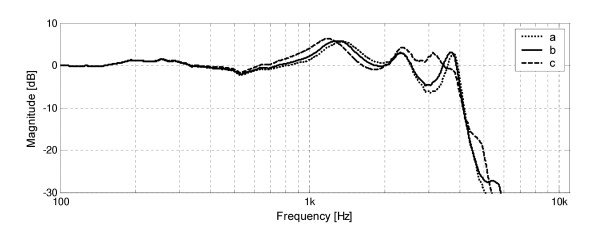
Frequency responses of the waveguide model of earmold system, obtained in computer simulations for varying earmold canal length: a) 2 mm, b) 10 mm, c) 20 mm.

The results of experiments are presented in a form of transfer function plots (magnitude expressed in decibels vs. frequency plotted on a logarithmic scale). Fig. 2 shows transfer function plots of the examined model for varying earmold canal length. Several resonances (local maxima) are visible in the frequency response plots. The first (main) resonance is located around 1 kHz and has the highest amplitude. There are two more significant resonances: first in the 2 – 3 kHz range, second in the 3 – 4 kHz range, both have lower amplitude than the main resonance. Increasing the earmold canal length shifts all resonances towards lower frequencies and slightly increases amplitude of each resonance. The difference between plots for earmold canal length 2 mm and 10 mm is small. However, increasing earmold length from 10 mm to 20 mm caused significant amplification in 2–4 kHz range. Modifying the earmold canal length does not affect frequency response above 4 kHz (where the receiver significantly attenuates the signal) and below 750 Hz.

**Figure 3:**
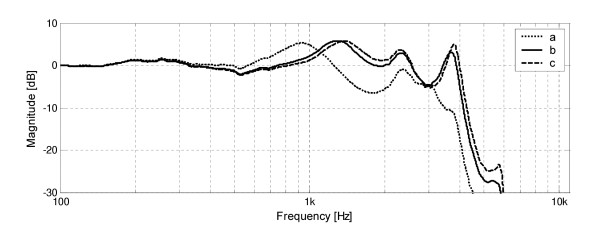
Frequency responses of the waveguide model of earmold system, obtained in computer simulations for varying earmold canal diameter: a) 1.1 mm, b) 2.4 mm, c) 4.0 mm.

The results of a similar experiment in which the diameter of the earmold canal was altered, is presented in Fig. 3. It is evident that a small diameter of earmold canal (1.1 mm) largely reduces the amplitude of transfer function for all frequencies above 1 kHz. Increasing the earmold diameter to 2.4 mm improves amplification in this range, but further increase in diameter gives much less increase in the amplitude level. The increase in the earmold canal diameter is accompanied by shifting of the resonances towards higher frequencies.

In another experiment, the diameter of the tubing was changed. This modification alters frequency response only in 1–3 kHz range. Increasing the tubing diameter causes increase in amplitude of the resonances (most evident for the first resonance) and small increase in resonant frequencies. The length of the tubing is not modified in practical applications. In another case that was studied, a single cylindrical tube for the earmold canal section was replaced by a set of three cylindrical tubes of the increasing diameter. This modification caused a small increase in amplitude above 3 kHz. However, due to attenuation caused by frequency response of the receiver, this change is not significant.

**Figure 4:**
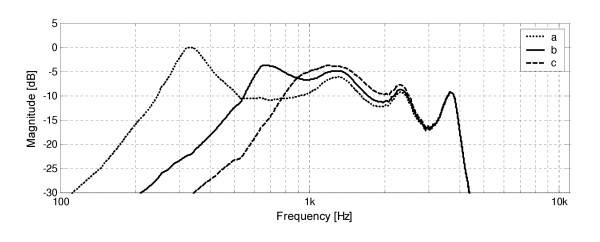
Frequency responses of the waveguide model of earmold system with the vent, obtained in computer simulations for varying vent diameter: a) 1 mm, b) 2 mm, c) 3 mm)

The influence of the vent of different length and diameter on frequency response of the model was also examined. Results of this experiment shown in Fig. 4 prove that including the vent in the model caused, as expected, significant attenuation for low frequencies – the model becomes a band-pass filter. Comparing plots of frequency responses of the model with and without vent, it can be observed that a new resonance occurs around the lower cut-off frequency. Decreasing the vent diameter result in shift of the lower cut-off frequency and resonances (in the range up to 2.5 kHz) towards lower frequencies. Also, increase in the amplitude of the resonance located at the lower cut-off frequency and decrease in the amplitude of the remaining resonances can be observed.

Comparison of the results of computer simulations with the measurement data, published by earmold system manufacturers, showed that the same changes in frequency responses caused by the change of length or diameter of a selected waveguide section, are observed in both measurement data of a real earmold system and in computed model responses. Therefore, it may be concluded that the developed computer model of the hearing aid earmold system properly simulates the real earmold system behavior, with sufficient accuracy.

## Conclusion

The waveguide modeling method allowed the authors to develop a computer model of the BTE hearing aid earmold system. It is possible to represent the earmold system as a set of cylindrical tubes, which is easily modeled using the waveguide method. The waveguide elements (main earmold system and vents) are modeled using delay lines and scattering junctions, while other factors (receiver response, ear canal and eardrum) are simulated either by digital filters in the waveguide model or by separate blocks.

The developed computer model of the earmold system was implemented using the MATLAB computer system. The results of experiments performed using the described model proved that the model behaves similarly to the real earmold system. Therefore, it may be concluded that waveguide modeling method, which has been so far applied almost exclusively to the synthesis of musical instruments, is a valuable tool for analysis of other acoustical systems, such as described hearing aid earmold system. The main advantage of the waveguide modeling method, apart from its easy and efficient implementation in a computer, is its straightforward relationship between real system parameters (length, diameter) and model parameters (delays, reflection coefficients). The main disadvantage of this method is problematic simulation of non-linear and frequency-dependent factors that cause energy losses in the acoustic system.

The experiments described in this paper, performed using the model developed, helped authors to propose a computer system for designing and evaluation of the hearing aid earmold system. Such a system may be useful in the process of hearing aid fitting. Based on the computer simulations, one will be able to compare the acoustical properties of various earmold systems, to change the model parameters until adequate frequency characteristics are obtained and then to use the simulation results to create the earmold system of a hearing aid optimally fitted to the needs of a hearing impaired person. The proposed system is not intended to replace the physicians, but to optimize their work by providing fast and efficient method of designing the earmold system of a hearing aid.

## Competing interests

The author(s) declare that they have no competing interests.

## Authors' contributions

Both authors contributed equally to this work. Both authors read and approved the final manuscript.
